# Modelling how negative plant–soil feedbacks across life stages affect the spatial patterning of trees

**DOI:** 10.1038/s41598-023-44867-0

**Published:** 2023-11-05

**Authors:** Annalisa Iuorio, Maarten B. Eppinga, Mara Baudena, Frits Veerman, Max Rietkerk, Francesco Giannino

**Affiliations:** 1 Present Address: Department of Engineering, Centro Direzionale-Isola C4, Parthenope University of Naples, 80143 Naples, Italy; 2 Faculty of Mathematics, University of Vienna, Oskar-Morgenstern-Platz 1, 1090 Vienna, Austria; 3 Department of Geography, University of Zurich, Winterthurerstrasse 190, 8057 Zurich, Switzerland; 4 Environmental Sciences Group, Copernicus Institute of Sustainable Development, Utrecht University, 3508 TC, Utrecht, The Netherlands; 5 Institute of Atmospheric Sciences and Climate (CNR-ISAC), National Research Council of Italy, Corso Fiume 4, 10133 Torino, Italy; 6 Mathematical Institute, Leiden University, Niels Bohrweg 1, 2300 RA Leiden, The Netherlands; 7 Department of Agricultural Sciences, University of Naples Federico II, via Università 100, 80055 Portici, Italy

**Keywords:** Ecological modelling, Theoretical ecology, Applied mathematics

## Abstract

In this work, we theoretically explore how litter decomposition processes and soil-borne pathogens contribute to negative plant–soil feedbacks, in particular in transient and stable spatial organisation of tropical forest trees and seedlings known as Janzen-Connell distributions. By considering soil-borne pathogens and autotoxicity both separately and in combination in a phenomenological model, we can study how both factors may affect transient dynamics and emerging Janzen–Connell distributions. We also identify parameter regimes associated with different long-term behaviours. Moreover, we compare how the strength of negative plant–soil feedbacks was mediated by tree germination and growth strategies, using a combination of analytical approaches and numerical simulations. Our interdisciplinary investigation, motivated by an ecological question, allows us to construct important links between local feedbacks, spatial self-organisation, and community assembly. Our model analyses contribute to understanding the drivers of biodiversity in tropical ecosystems, by disentangling the abilities of two potential mechanisms to generate Janzen-Connell distributions. Furthermore, our theoretical results may help guiding future field data analyses by identifying spatial signatures in adult tree and seedling distribution data that may reflect the presence of particular plant–soil feedback mechanisms.

## Introduction

A key challenge in ecology is understanding the large diversity of plant species that coexist within communities. Classical ecological theory, often developed with Lotka-Volterra type models, has clearly shown that more species can coexist in communities where species experience stronger negative conspecific density dependence^1–4^. However, due to the relatively abstract nature of these models, it remains difficult to link the strength of conspecific density dependence to different mechanisms that can be observed and quantified in the field. The development of more detailed theoretical frameworks, involving differential and possibly intertwined mechanisms, may improve our ability to connect ecological theory of density dependence and the maintenance of diversity to empirical field observations^5–8^.

In this context, a promising approach is the development of spatially explicit models, which can generate hypotheses on the ways in which density-dependent mechanisms would be reflected in the spatial patterning of plant communities^9,10^. In tropical forest tree communities, for example, it has been well established since the classical observations by Janzen (1970) and Connell (1971) that the spatial distribution of seedlings is markedly different from the spatial distribution of seeds. Typically, conspecific seedling density is highest at intermediate distances from the parent tree^11–14^.

While early studies often focused on explaining Janzen-Connell (JC) distributions by herbivory^15–17^, evidence is accumulating that negative plant–soil feedbacks can play an important role in this process as well (^12,18–20^ but see^21^). Here, plant–soil feedback refers to a two-step process in which the plants mediate their local soil environment, which in turn affects the fitness of this host plant and the surrounding plant individuals. In forests, this effect is expected to mainly impact younger individuals that may not be able to survive in the soil environment created by the presence of conspecific adults^22–24^. Studies utilizing greenhouse experiments, field experiments, forest census data and meta-analyses of plant–soil feedback experiments have identified the accumulation of species-specific soil pathogens as a likely mechanism generating negative plant–soil negative feedback^12,13,20,25,26^. However, alternative mechanisms generating negative plant–soil feedback have also been suggested. Specifically, accumulation of conspecific DNA fragments may create an auto-toxic soil environment^27^, which prevents seed germination and growth^28^. In a recent review on the mechanisms of vegetation pattern formation by plant–soil feedbacks, Inderjit et al^29^ also discuss the role of autotoxicity. Among the putative mechanisms for autotoxicity, the inhibitory and toxic effect of extracellular self-DNA produced by litter decomposition has been widely unnoticed in this context and should be highlighted^30^. In two papers, Mazzoleni et al.^27,31^ reported on the discovery that fragmented extracellular self-DNA (i.e. DNA originating from conspecifics) produces species-specific inhibitory effects in plants. Mazzoleni et al.^31^ described experimental observations of autotoxicity, i.e., species-specific inhibition on seedling root growth of several species by their own decomposed litter. Moreover, laboratory experiments confirmed the inhibitory effect of purified conspecific DNA on seed germination and root growth only when the treatments were performed using fragmented DNA. The occurrence of the self-DNA inhibitory effect was further generalized testing several taxa such as bacteria, protozoa, algae, fungi, and insects^32^. This mechanism might be expected to affect trees in an earlier life stage than the pathogen accumulation mechanism described above; while it has not been observed in tropical forest yet, it would be interesting to theoretically explore how the presence of this mechanism may differ from or interact with forest community dynamics as generated by pathogen accumulation.

The above description points to another shortcoming of classic Lotka–Volterra type models, in that they typically do not distinguish between mechanisms acting at different times or life stages in the population(s) of interest. However, studying the emergence of Janzen–Connell distributions would, besides explicit consideration of space, require explicit consideration of (life)time as well, as the recognition of the spatial patterns of interest requires differentiation between seeds, seedlings and adults. Such explicit modelling frameworks can then also address the question of how different mechanisms may contribute to driving Janzen-Connell distributions, depending on the timing of their effects within the plants’ lifecycle^33,34^.

Here, we simulate the transient dynamics of conspecific seed, seedling and adult tree distributions, using a recently developed spatially explicit, stage-structured population model^35^. Parameterising the model using data from tropical forest ecosystems, and assuming that both the soil pathogens and DNA toxicity mechanisms may operate simultaneously in tropical forests, our aim is to assess how these different mechanisms generating negative plant–soil feedback affect these spatial distributions and compare their effects. In addition, we were interested in how these distributions may be mediated by the outcomes of evolutionary processes leading to particular plant strategies. Specifically, we considered tree species’ dispersal ability and growth-defence tradeoffs, where it is hypothesized that during forest succession, the average position of the species present along both these axes will be shifting^36–38^. Our analyses were focused around answering the following research questions: (1) How do emergent spatial patterns of adult trees and tree seedlings depend on the specific mechanism impacting the seedlings? (2) To what extent does the dispersal ability of tree species moderate the spatial patterns of adult and seedlings? (3) To what extent are plant strategies along the growth-defence trade-off reflected in the spatial patterns of adult and seedlings?

## Methods

### Mathematical model

**Figure 1:**
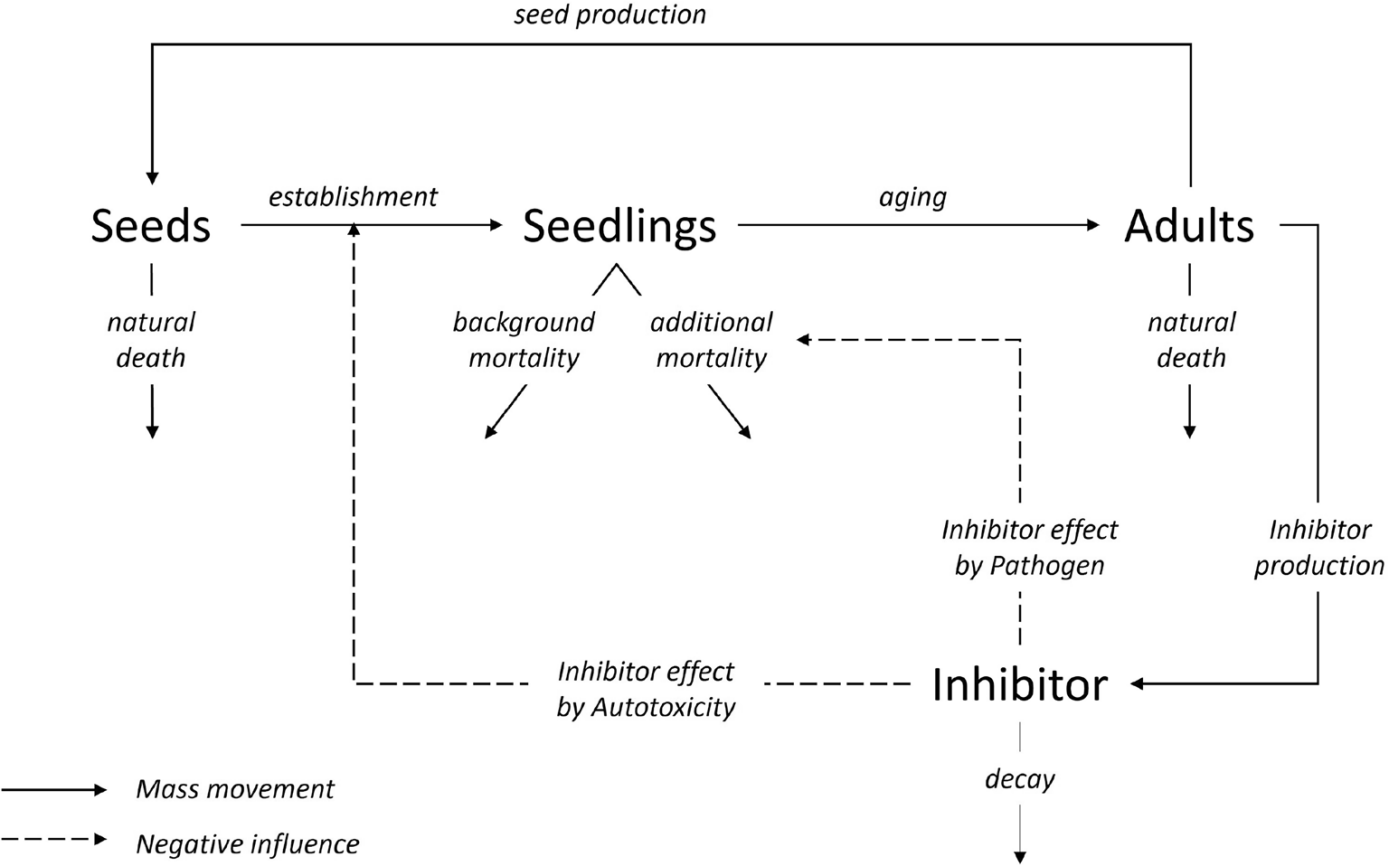
Schematisation of the dynamics between seeds *S*, seedlings *N*, adults *A*, and toxicity *I* as described by Eq. (1). Continuous lines represent transition of mass, whereas dashed lines indicate negative influences, which generate density-dependent feedbacks.

In our framework, plant–soil negative feedback (NF) manifests itself both during the seed-to-seedling transition (in terms of *growth inhibition*) and at the seedlings life-stage (in terms of *increased mortality*). The first effect can be often attributed to the presence of extracellular self-DNA (also known as autotoxicity), whereas the second effect is mainly linked to soil-borne pathogens. We base our investigation on the model recently introduced in^35^ which consists of partial differential equations (PDEs) given by

1 $$\begin{aligned} \begin{aligned} \frac{\partial S}{\partial t}&= d_S \cdot \Delta S + g_S \cdot A - k_S \cdot S, \\ \frac{\partial N}{\partial t}&= \frac{g_N \cdot S}{1+\beta \cdot e^{r_T \cdot I}} - \left( k_N + g_A \left( 1-\frac{A}{A_{\rm{max}}} \right) + r_P \cdot I \right) \cdot N, \\ \frac{\partial A}{\partial t}&= \left( g_A \cdot N + c_A \cdot A \right) \cdot \left( 1-\frac{A}{A_{\rm{max}}} \right) - k_A \cdot A, \\ \frac{\partial I}{\partial t}&= d_I \cdot \Delta I + c_T \cdot A - k_I \cdot I, \end{aligned} \end{aligned}$$

which we here briefly describe. Vegetation is considered in terms of biomass and is divided into three compartments, using an age-structure modelling approach^39^, corresponding to three different life-stages, namely seeds *S* (${\textrm kg}/{\textrm m}^2$), seedlings *N* (${\textrm kg}/{\textrm m}^2$) and adults *A* (${\textrm kg}/{\textrm m}^2$). This is the minimum number of life stages that needs to be considered for the study of the JC distributions described above. Furthermore, the general inhibitor variable *I* (${\textrm kg}/{\textrm m}^2$) mimics the accumulation of both autoxicity and pathogens. The former is reflected in the growth inhibition term, while the latter is reflected in an additional mortality term. The relationships between the four model state variables at any spatial point ${\textbf{x}}=(x,y)$ and any time *t* are schematically represented in Fig. 1: the increase of seeds’ density is influenced by adult tree production via the per capita seed production rate $g_S$ and seed dispersal $d_S$, whereas their natural decay rate (including predation) is represented by $k_S$. Seeds then germinate and the seedlings might establish or not, depending also on the inhibitor due to the effect of autotoxicity via the function $\frac{g_N \cdot S}{1+\beta \cdot e^{r_T \cdot I}}$. Seedlings have a background mortality rate $k_N$, with additional mortality induced by pathogens via the term $r_P \cdot I$. The seedlings which survive then grow into the next life stage according to the function $g_A \left( 1-\frac{A}{A_{\rm{max}}} \right)$. This function implies that under near-closed canopies, seedling growth becomes very small, may remain in this life stage for extensive periods of time (e.g.^40^). Adults’ density grows logistically because of seedlings transitioning to the adult stage at rate $g_A$ and intrinsic growth $c_A$/mortality $k_A$. The inhibitor density grows due to the presence of adult trees (constituting the vast majority of biomass in the system, e.g.^41^) at a rate $c_T$, decays naturally at a rate $k_I$, and diffuses in the soil at a rate determined by the coefficient the coefficient $d_I$. In this framework, links to the Janzen-Connell hypothesis can be found in transient patterns where a ring of seedlings emerges around the adult tree (whose density is concentrated in the centre of the ring). Mathematically, this consists in travelling wave solutions, whose existence has been analysed in^35^. From here on, we refer to this phenomenon of transient, spatially localised ring patterns of seedling density as the Janzen-Connell distribution. The (homogeneous) steady-states associated to System (1) are provided in “Appendix B”.

Values and meaning of the non-negative model parameters in (1) are provided in Table 1. We used the findings of previous empirical ecological studies of tropical forests to obtain order-of-magnitude realistic estimates of the model parameters, as explained in “Appendix A”.

**Table 1:** Description, values, and units for model parameters in System (1), obtained through parameterisation and calibration (see “Appendix A”).

Parameter	Description	Values	Units
$g_S$	Growth rate of *S*	$6.67 \times 10^{-8}$–0.033	${\textrm year}^{-1}$
$k_S$	*S* turnover rate	0.33–0.5	${\textrm year}^{-1}$
$g_N$	Transition rate from *S* to *N*	0.25–25	${\textrm year}^{-1}$
$\beta$	Establishment sensitivity to toxicity parameter	$10^{-5}$	–
$r_T$	Establishment sensitivity to toxicity parameter	0–68	${\textrm m}^2 \, {\textrm kg}^{-1}$
$k_N$	Death rate of *N*	0.02–0.74	${\textrm year}^{-1}$
$r_P$	Increased mortality of *N* caused by *I*	0–2	${\textrm m}^2 \, {\textrm kg}^{-1} \, {\textrm year}^{-1}$
$g_A$	Transition rate from *N* to *A*	0.02-100	${\textrm year}^{-1}$
$c_A$	Growth rate in *A*’s biomass density	0.25	${\textrm year}^{-1}$
$A_{\rm{max}}$	Maximum capacity for *A*	30	${\textrm kg} \, {\textrm m}^{-2}$
$k_A$	Mortality rate of *A*	0.01	${\textrm year}^{-1}$
$c_T$	Growth rate of *I* due to *A*	1	${\textrm year}^{-1}$
$k_I$	Toxicity decay rate	0.7	${\textrm year}^{-1}$
$d_S$	Diffusion coefficient for *S*	0–4	${\textrm m}^2 \, {\textrm year}^{-1}$
$d_I$	Diffusion coefficient for *I*	0–10	${\textrm m}^2 \, {\textrm year}^{-1}$

### Numerical setup

In order to analyse the emergence of Janzen–Connell distributions for seedlings around their parent tree, we numerically investigate Eq. (1) on a square bounded domain $\Omega \in {\mathbb {R}}^2$ of edge length *L* with no-flux boundary conditions and an initial number of seeds distributed over a patch in the centre of the domain, i.e. 

2a $$\begin{aligned} S({\textbf{x}},0)=S_0({\textbf{x}}), \quad N({\textbf{x}},0)=0, \quad A({\textbf{x}},0)=0, \quad I({\textbf{x}},0)=0, \quad {\textbf{x}} \in \Omega , \end{aligned}$$

2b $$\begin{aligned} \partial _n S=0, \quad \partial _n N=0, \quad \partial _n A=0, \quad \partial _n I=0, \quad {\textbf{x}} \in \partial \Omega , \quad t \ge 0. \end{aligned}$$

Here $\partial \Omega$ is the boundary of $\Omega$, $\partial _n$ is the normal derivative on $\partial \Omega$, ${\textbf{x}}=(x,y)$, and $S_0$ corresponds to an initial seed distribution.

Our numerical setup is defined in Matlab on a square lattice of $m \times m$ elements—with $m=600$—discretized with a spatial grid of $\delta x=\delta y=0.1$ meters. The total simulation time is ${\mathscr {T}}=80$ years with timesteps of $\delta t=0.001$ years. During this time, we assume that there are no major disturbances in the system, such as forest gap formation. This is a simplification, as such disturbances may determine spatial patterns of adult and seedling distributions^42^. Following^43^, the numerical scheme used in our simulations is based on a forward Euler integration of the finite-difference equations obtained by discretizing the diffusion operator with no-flux (i.e. Neumann) boundary conditions. These boundary conditions represent the scenario where no outward biomass flow occurs in the considered domain. Alternative boundary conditions (e.g. periodic) could also be taken into account; this would, however, not substantially affect the numerical results shown and discussed below. Since our main goal is to understand the temporal evolution of the Janzen–Connell distribution as well as its relation with the main systems’ parameters, the initial condition for *S* is kept the same in all simulations. Specifically, the initial distribution of *S* consists of a symmetric concentration at the centre of the domain representing a circular patch of seeds, namely

3 $$\begin{aligned} S_0(x,y):= e^{-\frac{L^2-2 L (x+y)+2 \left( x^2+y^2\right) }{2 L \delta x}} \end{aligned}$$

In all simulations, the parameters $\beta$, $c_A$, $A_{\rm{max}}$, $k_A$, $c_T$, and $k_I$ are assigned to the unique values listed in Table 1. Moreover, according to parameter values within the range observed for tropical tree species in previous empirical studies, we fix $g_S=0.033\, {\textrm year}^{-1}$, $k_S=0.33\, {\textrm year}^{-1}$, $g_N=5\, {\textrm year}^{-1}$, $k_N=0.5\, {\textrm year}^{-1}$, and $d_I=0.5\, {\textrm m}^2 \, {\textrm year}^{-1}$ (see “Appendix A” for details). The influence of the remaining parameters $r_T$, $r_P$, $g_A$, and $d_S$ on the Janzen-Connell distribution is analysed in more detail in “Results”.

Given the symmetry property of our simulated patterns, we consider 1D sections of the 2D numerical profiles, in particular we focus our attention on $x \in \left[ \frac{L}{2}, \, L \right]$. This in fact allows us to better visualise the impact of the individual factors on the shape of the Janzen–Connell distribution. Since the main effects are visible on the seedlings profiles, we focus our attention on the profiles for the seedlings’ density distribution and show the corresponding profiles for the other variables *S*, *A*, and *I* in “Appendix C”.

### Analyses

In this section, we introduce the setup leading to the investigation of Janzen-Connell distributions as transient patterns as well as of the impact of different effects (represented by selected parameters in the model) on their emergence. In particular, we analyse the influence of negative plant–soil feedbacks via growth inhibition and increased mortality on these structures, by considering these two effects separately. Subsequently, we use our numerical framework to perform two simulation experiments with the aim to assess how changes in seed dispersal and growth/defence strategies affect the features of the Janzen–Connell distributions.

**Figure 2:**
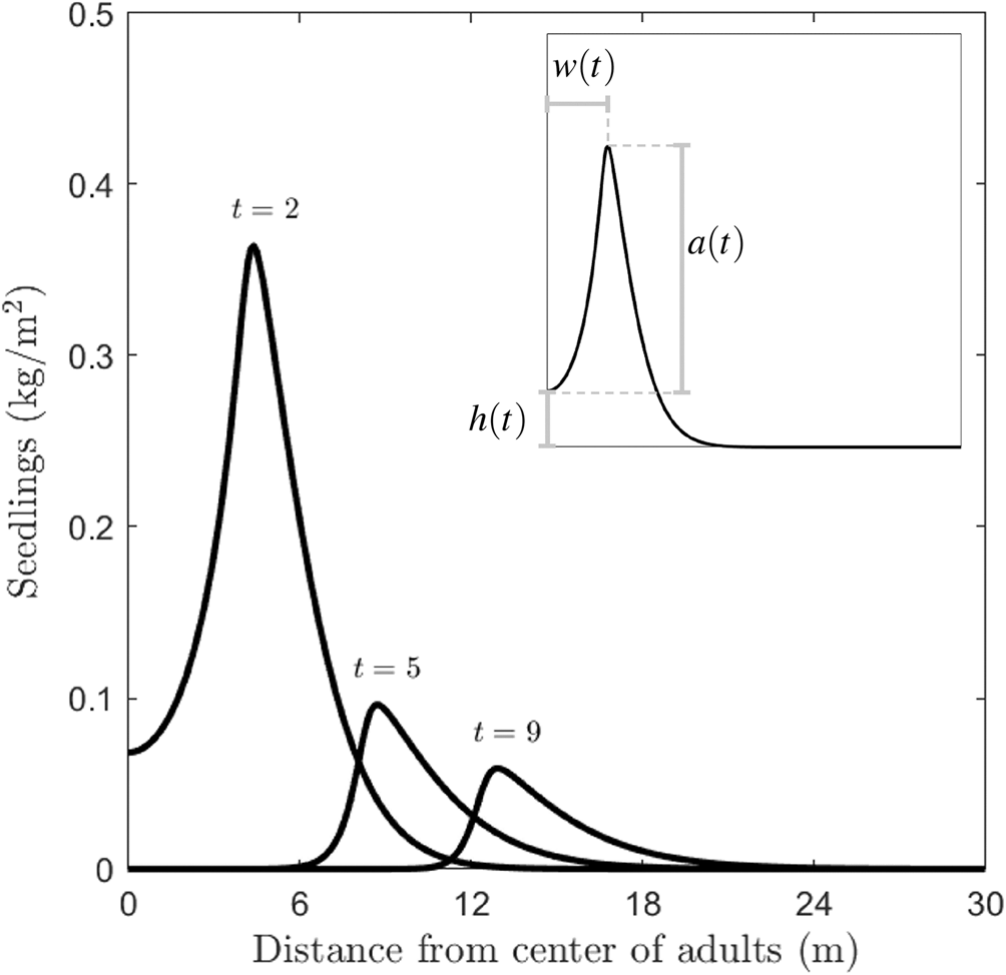
Time evolution of a typical profile for the seedling density *N* (solid line) at different simulation times, as indicated by the corresponding labels. In the inset plot, a schematic representation of the width *w*(*t*) and amplitude *a*(*t*) is displayed, corresponding to the *N* numerical profile at $t=2$ (see Eqs. (4) and (5), respectively). Based on this definition, we see that as *t* increases *a*(*t*) decreases and *w*(*t*) increases.

To this aim, we first introduce three indices for the *N* profile, namely *distance* (*w*(*t*)), *amplitude* (*a*(*t*)), and *centre height* (*h*(*t*)), measuring the following features of the seedlings’ biomass gap in the centre of the domain (i.e. under the parent tree) (see Fig. 2):

- The *distance* of the pattern at time *t* is defined as the distance between the point $x_{{{{\textrm max}}}(N)}$ in the right half-domain where the maximum of the *N* biomass is reached and the centre of the gap (i.e. of the spatial domain), corresponding to
  4 $$\begin{aligned} w(t):= \left|{{\textrm argmax}}_{x \in [0,L]}(N(t,x))- \frac{L}{2} \right|. \end{aligned}$$
- The *amplitude* of the pattern at time *t* is defined as the difference between the maximum value of seedling biomass density *N* and its value at the centre of the gap, i.e.
  5 $$\begin{aligned} a(t):= \left|{{\textrm max}}(N(t,x)) - N\left( t,\frac{L}{2}\right) \right|. \end{aligned}$$
- The *centre height* of the pattern at time *t* is defined as the value of seedling biomass density *N* at the centre of the gap, i.e.
  6 $$\begin{aligned} h(t):= N\left( t,\frac{L}{2}\right) . \end{aligned}$$

In the following analyses, the profiles considered for our comparison of different parameter regimes are calculated at the time $t_{\rm{max}}$ where the *N* maximum amplitude is reached, i.e.

$$\begin{aligned} t_{\rm{max}}:= {{\textrm argmax}}_{t \in [0, \, {\mathscr {T}}]}(a(t)). \end{aligned}$$

In the general setup, in addition to the parameters assuming a fixed value in Table 1, we consider $r_T=34 \, {\textrm m}^2 \, {\textrm kg}^{-1}$, $r_P=1 \, {\textrm m}^2 \, {\textrm kg}^{-1} \, {\textrm year}^{-1}$, $g_A=0.2 \, {\textrm year}^{-1}$, and $d_S=3 \, \, {\textrm m}^2 \, {\textrm kg}^{-1}$. Some of these values might vary when focusing on specific scenarios, as described below.

#### Inhibition

To answer our first research question, we consider two simulation scenarios, where the inhibition of seed establishment and the increase of the seedlings’ mortality rate—both effects induced by the inhibitor *I*—are analysed individually: first, we only consider growth inhibition (setting $r_P=0$), and then only increased mortality (by fixing $r_T=0$). In this study, we associate seed decay mostly with autotoxicity while seedling mortality is mostly attributed to soil pathogens. Here we chose to represent a tree species with light seeds that disperse rather far ($d_S=3$ ${\textrm m}^2 \, {\textrm year}^{-1}$, $g_A=0.2 \, {\textrm year}^{-1}$).

In order to consider different intensities of both effects, we set $r_T^{{\textrm ref}}=68 \, {\textrm m}^2 \, {\textrm kg}^{-1}$ and $r_P^{{\textrm ref}}=2 \, {\textrm m}^2 \, {\textrm kg}^{-1} \, {\textrm year}^{-1}$, i.e. the maximum values in their respective feasibility range as shown in Table 1, and compare the *N* profiles of maximum amplitude for $25\%, \, 50\%, \, 75\%$, and $100\%$ of $r_T^{{\textrm ref}}$, $r_P^{{\textrm ref}}$, respectively. In order to test the robustness of our outcome, we perform a sensitivity analysis of the indices *a*(*t*), *w*(*t*), and *h*(*t*) at time $t={\mathscr {T}}$ in the two regimes mentioned above: first we fix $r_P=0$ and let $r_T$ vary within its range defined in Table 1, and then vice versa. A detailed description of the results of our numerical investigation is available in “Inhibition”.

#### Seed dispersal

To answer our second research question, we focus here on the effect of different dispersal rates. We thus assume that trees are influenced both by growth inhibition and increased mortality at a medium rate based on the parameter ranges in Table 1. Therefore, the corresponding parameters $r_T$ and $r_P$ are both fixed to $50\%$ of their reference value, i.e. $r_T=34 \, {\textrm m}^2 \, {\textrm kg}^{-1}$ and $r_P=1 \, {\textrm m}^2 \, {\textrm kg}^{-1} \, {\textrm year}^{-1}$, respectively. Here we also assume seeds are rather small ($g_A=0.2 \, {\textrm y}^{-1}$ as in “Numerical setup”). Introducing $d_S^{{\textrm ref}}=3 \, {\textrm m}^2 \, {\textrm year}^{-1}$, we compare the profiles for the state variables *S*, *N*, *A*, and *I* at $t=t_{\rm{max}}$ with different intensities of $d_S$, corresponding to $25\%, \, 50\%, \, 75\%$, and $100\%$ of $d_S^{{\textrm ref}}$. In particular, in addition to the one where both effects are assumed to have moderate impact on the system’s dynamics, we consider four additional situations given by the combination of low/high growth inhibition/increased mortality through the respective parameters $r_T$ and $r_P$, and analyse the impact of these combined effects on both distance and amplitude. We consider the following five scenarios corresponding to different magnitudes of $r_T$ and $r_P$:

(i) low $$r_T$$, low $$r_P$$,
(ii) low $r_T$, high $r_P$,
(iii) moderate $r_T$, moderate $r_P$,
(iv) high $r_T$, low $r_P$,
(v) high $$r_T$$, high $$r_P$$.

The results of our numerical investigation are discussed in “Seed dispersal”.

#### Growth/defence approaches

Finally, to answer our third and last research question, here we focus our attention on species moderately sensitive to growth inhibition (by fixing $r_T=47.6\, {\textrm m}^2 \, {\textrm kg}^{-1}$, i.e. $80\%$ of $r_T^{{\textrm ref}}$) and we analyse the impact of different approaches to growth and defence on the Janzen-Connell patterns. More precisely, we compare species which invest more resources in growth rather than in defence against the detrimental effect induced by pathogens, and vice versa. These two ecological scenarios are represented by a combination of transition rate from *N* to *A* and increased mortality rate either both high (i.e. $g_A=0.9 \, {\textrm y}^{-1}$ and $r_P=2 \, {\textrm m}^2 \, {\textrm kg}^{-1} \, {\textrm year}^{-1}$) or both low (i.e. $g_A=0.02 \, {\textrm y}^{-1}$ and $r_P=0.1 \, {\textrm m}^2 \, {\textrm kg}^{-1} \, {\textrm year}^{-1}$), respectively. The results of our numerical investigation are discussed in “Growth/defence approaches”.

## Results

**Figure 3:**
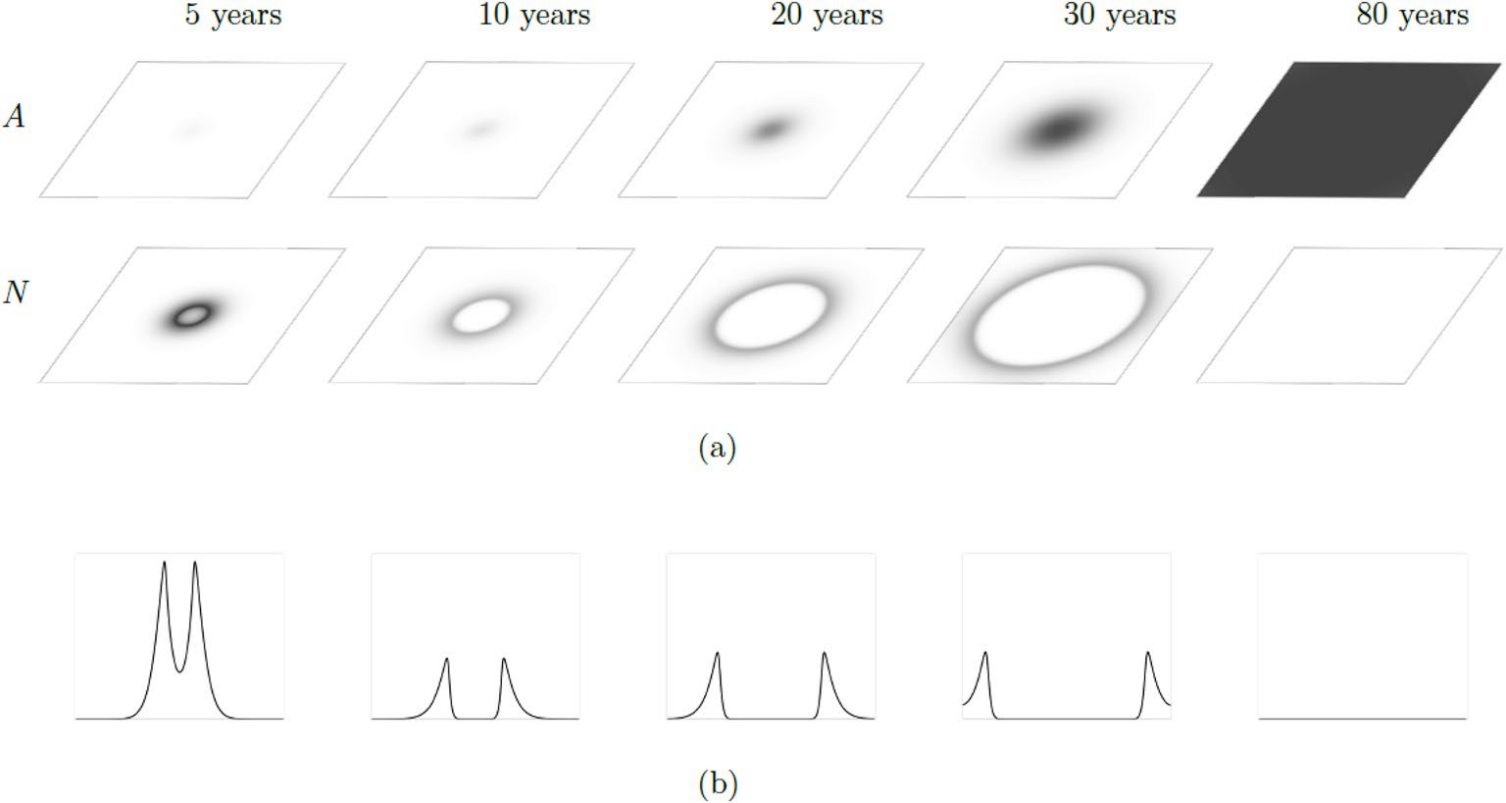
Spatio-temporal evolution of a prototypical Janzen–Connell distribution obtained by simulating System (1)–(2) on the two-dimensional bounded domain $\Omega$ for $t \in [0, \, {\mathscr {T}}]$. This transient pattern appears in the form a travelling wave, and the system converges to the uniform steady-state $E_1^*$ as $t \rightarrow \infty$. Darker areas represents higher biomass concentrations. (**a**) Dynamics of biomass densities for adults (*A*, upper panels) and seedlings (*N*, lower panels) between 5 and 80 years. (**b**) One-dimensional cross-sections of the corresponding two-dimensional profiles in (**a**) at $y=m/2$.

In this section, we provide the results related to the goals defined in “Analyses”. First, we focus on the temporal dynamics of the Janzen–Connell distribution as an emerging phenomenon, that may reveal itself in the transient spatial distribution dynamics of adults and seedlings. Our numerical investigation confirms the transient nature of the Janzen-Connell patterns analytically predicted in^35^, since the system exhibits travelling waves which, due to the finite dimension of the domain considered in our simulations, eventually converge to the stable steady-state $E^*_1$ as $t \rightarrow \infty$ for any considered parameter set. The five panels in Fig. 3 show the spatial distribution of seedlings *N* and adults *A* after 5, 10, 20, 30, and 80 years. Due to the influence of plant–soil negative feedback, seedlings reach their highest density at a suitable distance from the highest adults’ density—i.e. they are spatially arranged according to a Janzen–Connell distribution, In particular, also due to our assumption on the initial seeds distribution, symmetric, circular seedlings patterns develop around the adults concentration at the centre of the spatial domain. As $t \rightarrow \infty$, the system reaches a uniform configuration corresponding to the steady-state $E_1^*$.

### Inhibition

**Figure 4:**
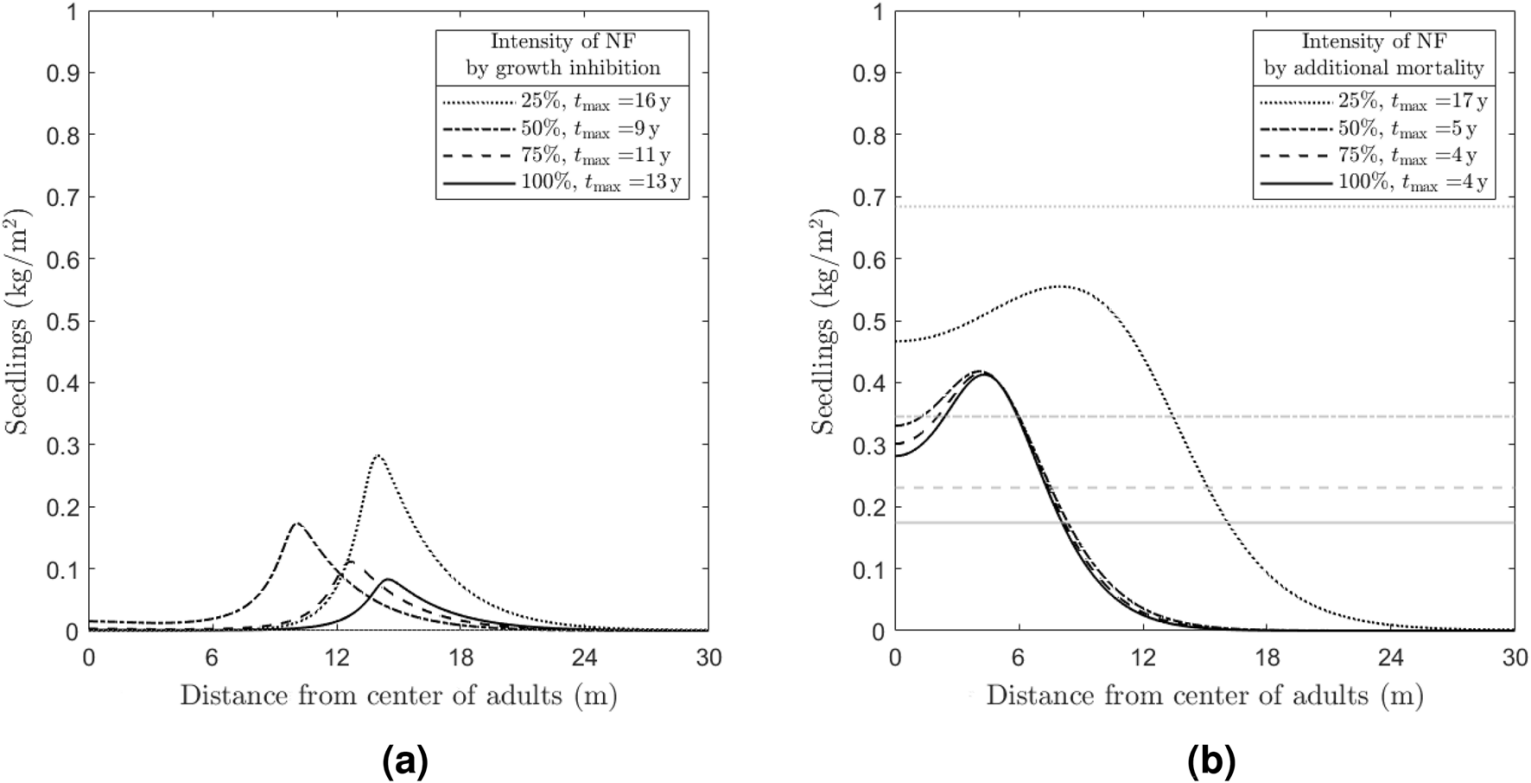
Numerical investigation of the NF influence on the emergence of Janzen-Connell distributions by means of exclusively growth inhibition (left panel) and increased mortality (right panel). The plots show the seedlings density (*N*) profiles at time $t=t_{\textrm max}$ (where the maximum amplitude is reached, indicated in the legend) obtained by simulating System (1) for (**a**) different values of the establishment sensitivity to autotoxicity parameter $r_T$, corresponding to different percentages of $r_T^{{\textrm ref}}=68 \, {\textrm m}^2 \, {\textrm kg}^{-1}$, and (**b**) different values of $r_P$ representing the increased mortality induced by soil-borne pathogens, corresponding to different percentages of $r_P^{{\textrm ref}}=2 \, {\textrm m}^2 \, {\textrm kg}^{-1} \, {\textrm year}^{-1}$ (the other parameter values are fixed as given in “Numerical setup”). The gray lines indicate the corresponding equilibrium $E_1^*$) reached by the system as $t \rightarrow \infty$; in panel (a), $N_1^*= 0$, whereas in (**b**) $N_1^*$ is proportional to $r_P$. For the profiles of the other state variables *S*, *A*, and *I*, see Fig. 8 in “Appendix C”.

We start by considering only the influence of plant–soil negative feedback by means of growth inhibition on the Janzen-Connell distribution, therefore neglecting the increased mortality effect by setting $r_P=0$ (see Fig. 4a). Here, we observe that the maximum value reached by *N* in correspondence of its maximum amplitude profile (i.e. ${{\textrm max}}(N(t_{\rm{max}},x))$) decreases as $r_T$ increases, coherently with the growth inhibition effect. Moreover—except for the case $r_T=25\% \, r_T^{{\textrm ref}}$—the times where the maximum amplitude profile occurs are directly proportional to $r_T$, i.e., smaller peaks appear later in time when growth inhibition is stronger, for large values of $r_T$. At low toxicity ($r_T=25\% \, r_T^{{\textrm ref}}$), on the other hand, the peak is higher and occurs later in time, i.e. does not obey the monotonicity rules outlined above. This suggests that low values of inhibitor, in the considered range, give the strongest JC pattern but take the longest time to appear, while large values of inhibitor give less evident patterns. Intuitively, for lower values of $r_T$ it takes longer for the seedlings’ density to form a depression in the centre and therefore reach a maximum amplitude profile. In all cases, System (1) converges to the stable steady-state $E^*_1$ as $t \rightarrow \infty$, where the *N* component is 0 (the expression for $E^*_1$ is provided in “Appendix B”).

Conversely, neglecting growth inhibition (by setting $r_T=0$) and assuming that the seedlings dynamics are influenced by the inhibitor only via increased mortality leads to a more pronounced depression and an inverse proportionality between $t_{\rm{max}}$ and $r_P$ (see Fig. 4b). This is coherent with ecological expectations, since a lower increased mortality coefficient implies that a higher amount of *N* biomass is needed in order to observe Janzen-Connell. In this scenario, the *N* component of the steady-state $E^*_{1}$ reached by System (1) as $t \rightarrow \infty$ is non-vanishing and decreases as $r_P$ increases.

Our simulations reveal that growth inhibition has a stronger effect on the emergence of Janzen-Connell distributions than increased mortality, possibly indicating that processes promoting seed decay may induce more distinct JC distributions than processes leading to increased mortality of seedlings (we note that the results shown in Fig. 4, in particular, are in agreement with^6^, Fig. 1(B)). On the other hand, the increased mortality effect induced by pathogens causes a reduced germination under the adult tree—a phenomenon slightly weaker than the occurrence of a Janzen–Connell distribution. Moreover, we observe that when both growth inhibition and increased mortality are neglected (i.e. $r_T=r_P=0$) no Janzen-Connell distribution emerges.

#### Sensitivity analysis of amplitude, distance, centre height

**Figure 5:**
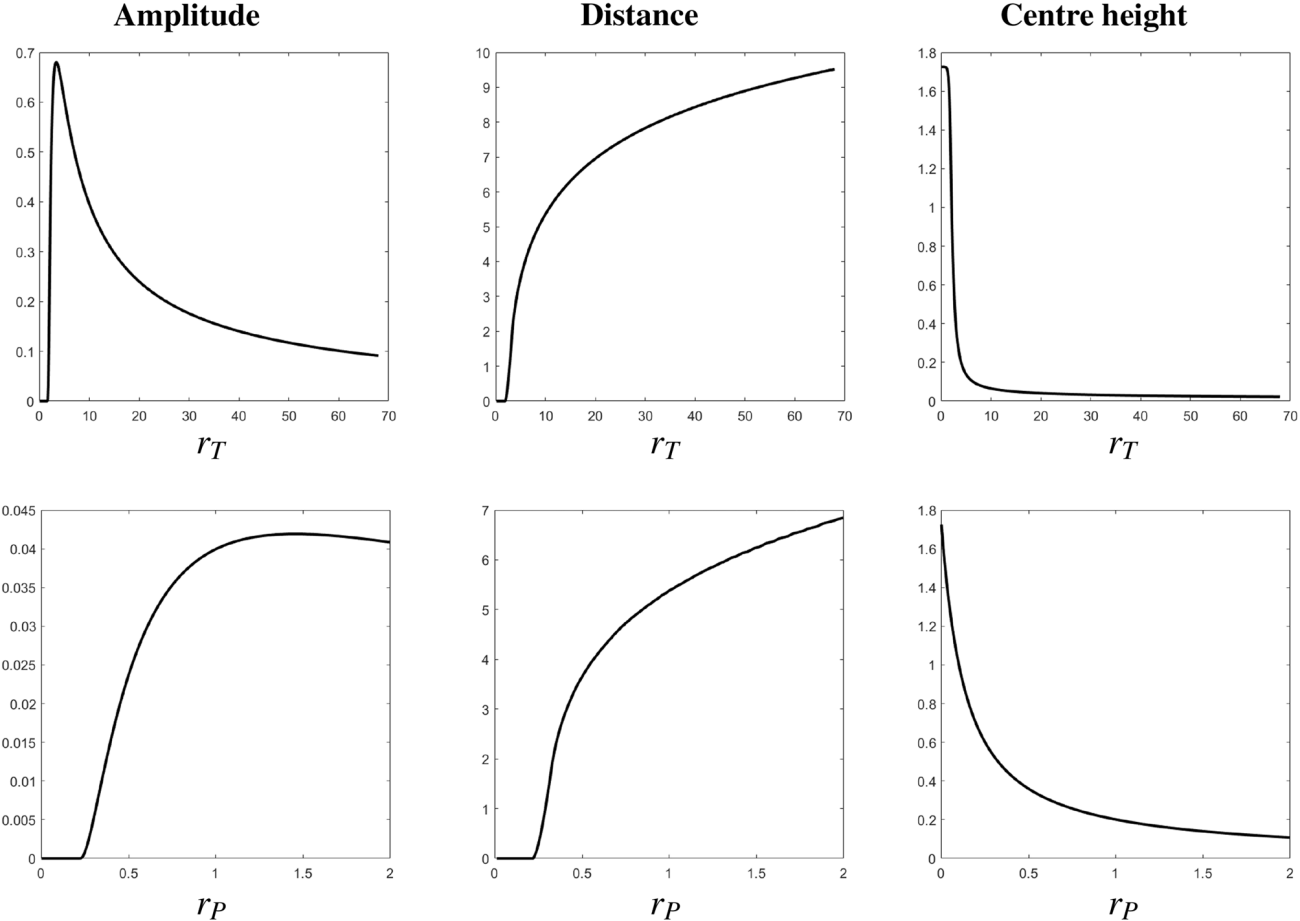
Amplitude *a*(*t*) (left column), distance *w*(*t*) (middle column), and centre height *h*(*t*) (right column) at $t={\mathscr {T}}$ as a function of $r_T$ with $r_P=0$ (top row) and as a function of $r_P$ with $r_T=0$ (bottom row). Other parameter values are fixed as in “Numerical setup”.

The results of our sensitivity analysis are summarised in Fig. 5. Comparing the amplitude, width and persistence for the two experiments where the two toxicity effects are represented separately, we observe that these three functions share some monotonicity properties in both scenarios; in particular, the amplitude $a({\mathscr {T}})$ is initially zero and then increases with $r_T$ up to a threshold value, above which it becomes a monotonically decreasing function. The distance $w({\mathscr {T}})$ also initially is close to zero and becomes monotonically increasing above a threshold value, remaining so in both cases. Finally, the centre height $h({\mathscr {T}})$ is approximately 1.8 and monotonically decreases in both scenarios. The distance and centre height function also reach values within the same order of magnitude, differently from the amplitude which in the case of $r_P=0$ is approximately ten times higher than in the case $r_T=0$. This indicates that, while both growth inhibition and increased mortality induce similar qualitative effects on the shape of the Janzen–Connell seedlings’ distributions, the quantitative properties of these transient patterns may significantly differ depending on which mechanism is prevailing in the underlying negative feedback.

### Seed dispersal

**Figure 6:**
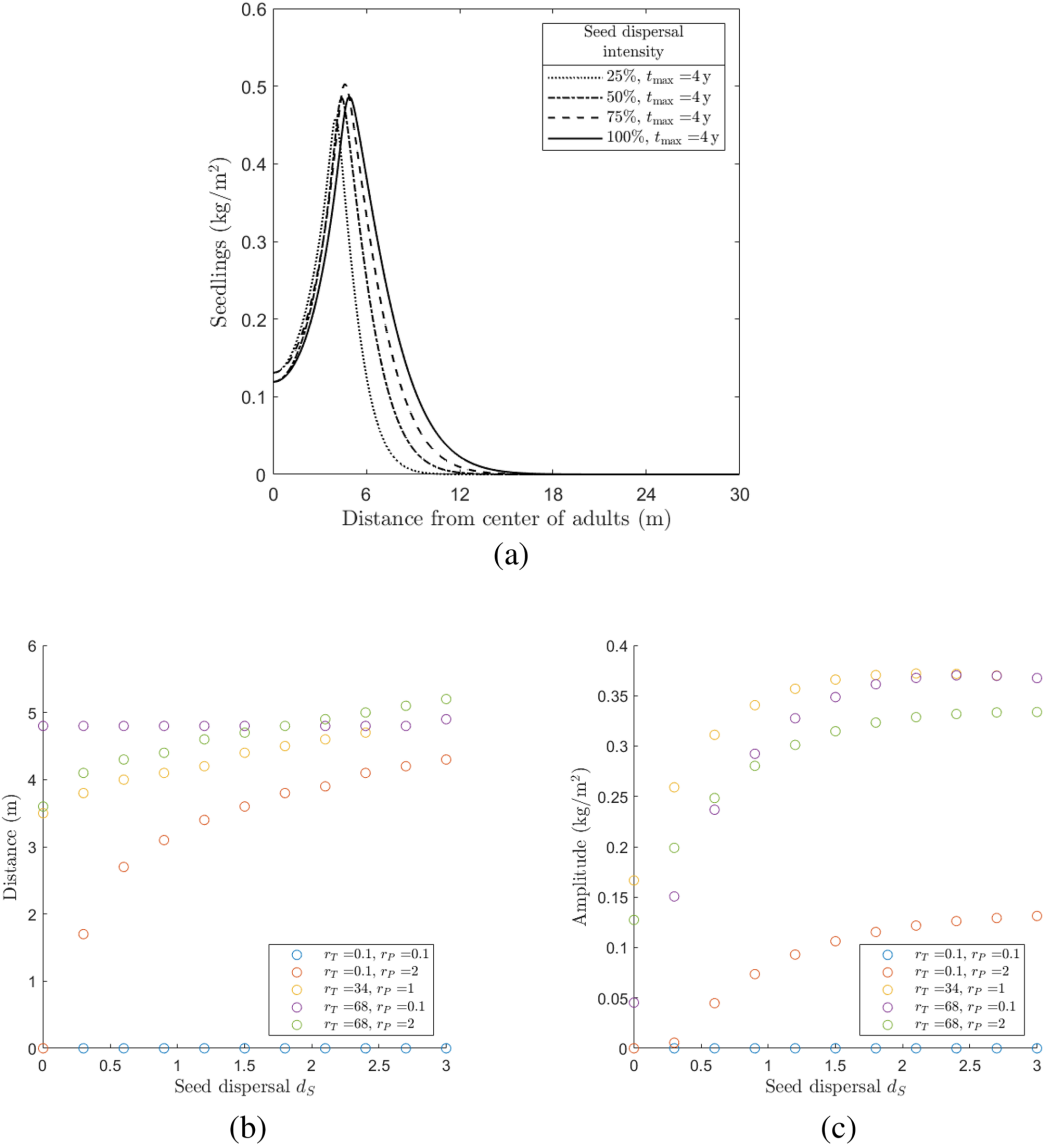
Numerical investigation of the seed dispersal influence on the emergence of Janzen–Connell distributions. (**a**) Seedlings density (*N*) profiles of maximum amplitude obtained by simulating System (1) for different values of the seed dispersal coefficient $d_S$, corresponding to different percentages of $d_S^{{\textrm ref}}=3 \, {\textrm m}^2 \, {\textrm year}^{-1}$ (the other parameter values are fixed as in “Numerical setup”). The values of the corresponding time $t_{\rm{max}}$ are indicated in the legend (for the profiles of the other state variables *S*, *A*, and *I*, see Fig. 9 in “Appendix C”). An investigation of distance and amplitude of the maximum amplitude profiles as functions of $d_S$ for five scenarios corresponding to different intensities of $r_T$ and $r_P$ is provided in (**b,c**), respectively.

The results of our numerical investigation shown in Fig. 6 can be summarized as follows. The time $t_{\rm{max}}$ (at which the maximum amplitude for *N* is reached) remains constant as the diffusion coefficient of the seeds $d_S$ varies, suggesting that this parameter does not play a crucial role in the realization of this profile. On the other hand, distance and amplitude seem to depend monotonically on $d_S$ (see Fig. 6a), which motivated us to extend our investigation to further interplay scenarios between growth inhibition and increased mortality effects. We retrieve indeed a monotonic trend of both distance (Fig. 6b) and amplitude (Fig. 6c) as functions of $d_S$, but the behaviour of this trend varies for the five different scenarios described in Section "Seed dispersal". In particular—except for scenario (i), where both functions remain approximately constant—distance is a monotonically increasing function of $d_S$ in scenarios (ii), (iii), (v) and has a mixed behaviour in scenario (iv), whereas amplitude is increasing in scenarios (ii), (v) and becomes decreasing after a threshold value for $d_S$ in scenarios (iii), (iv).

### Growth/defence approaches

**Figure 7:**
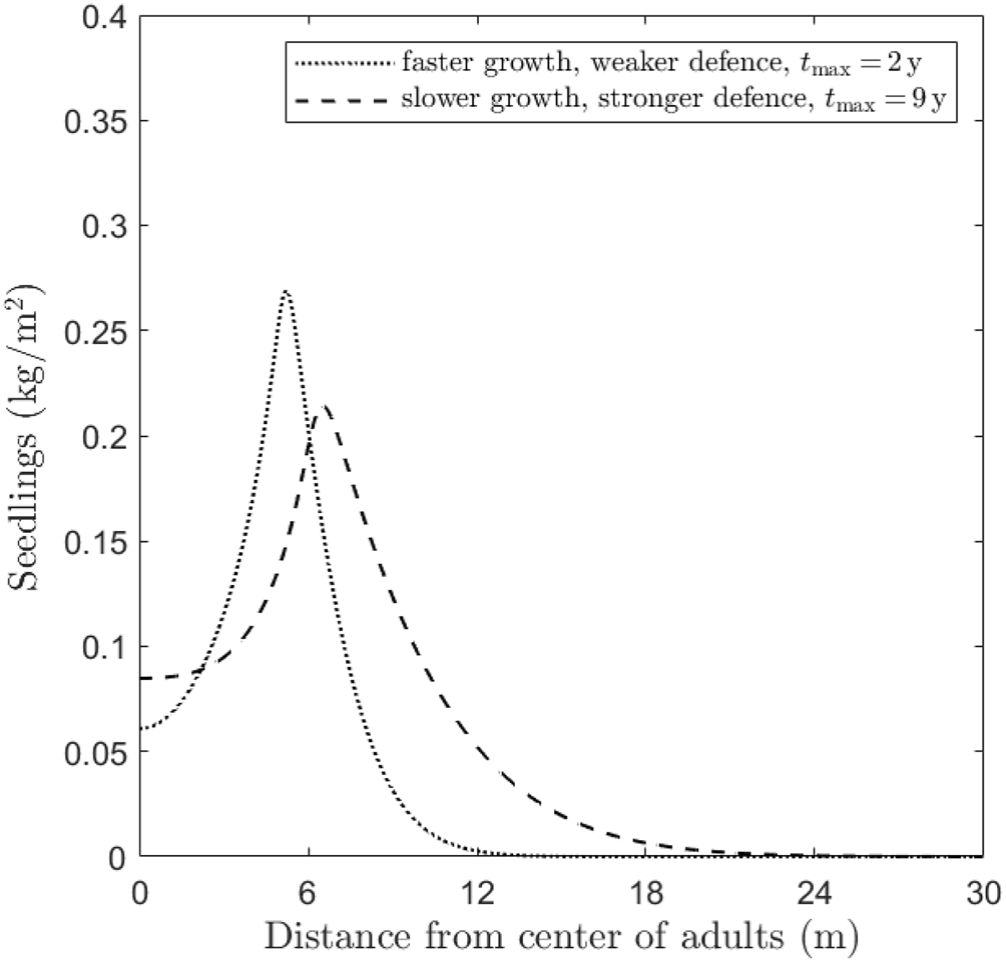
Numerical investigation of the influence of different growth/defence approaches on the emergence of Janzen–Connell distributions. The plots show the seedlings density (*N*) profiles at time $t=t_{\rm{max}}$ (where the maximum amplitude is reached, indicated in the legend), obtained by simulating System (1) for a species with faster growth, weaker defence ($g_A=0.9 \, {\textrm year}^{-1}$ and $r_P=2 {\textrm m}^2 \, {\textrm kg}^{-1} \, {\textrm year}^{-1}$, dotted line) and slower growth, stronger defence mechanisms ($g_A=0.02\, {\textrm year}^{-1}$ and $r_P=0.1 \, {\textrm m}^2 \, {\textrm kg}^{-1} \, {\textrm year}^{-1}$, dashed line), respectively (the other parameter values are fixed as in “Numerical setup”). For the profiles of the other state variables *S*, *A*, and *I*, see Fig. 10 in “Appendix C”.

Here, we compare two species with different growth vs defence strategies, to show how these impact the emerging Janzen-Connell patterns. We see that species which invest more in growth than in defence (corresponding to high $g_A$, high $r_P$) are able to reach a higher maximum value of the seedlings’ biomass than the ones which do the opposite (low $g_A$, low $r_P$), i.e. the amplitude index *a* is higher in the first case. However, the value of $t_{\rm{max}}$ in the first case is lower than the corresponding value in the second case, implying that plants which grow according to the second strategy—hence focusing their resources on fighting external detrimental factors—are more resilient. Coherently, while System (1) converges to $E_1^*$ in both cases as $t \rightarrow \infty$, our numerical investigation shows a faster convergence to this steady-state in the ecological scenario representing faster growth and weaker defence (see the corresponding *N* profiles in Fig. 7).

## Discussion

We have presented a model framework that links different types of negative plant–soil feedbacks to emergent spatial distribution patterns of seedlings around the parent tree. The two types of negative plant–soil feedback considered differ in the timing of the negative soil effects within the lifespan of the tree species. Empirical studies have shown how negative plant–soil feedbacks may involve a reduced germination of seeds (possibly due to the presence of soil autotoxicity, although pathogen also may have a role), as well as reduced survival of seedlings, attributed to the presence of soil-borne pathogens^11,12,23,28,44^. As our model framework explicitly considers the seed, seedling, and adult stages within a tree population, negative feedback through reduced seed germination and seedling survival can both be modelled (Fig. 1). By performing numerical simulations in which these two feedbacks operated either jointly or in isolation, our study clarified how operation of these two types of plant–soil feedback may be reflected in the spatial distribution patterns of tree seedlings around parent trees. Using this type of full-factorial modelling experiments is an important way in which theoretical studies can identify potential links between ecological patterns and underlying processes, which is a classical challenge in ecology^6,45–49^. Our metrics distance, amplitude, and centre height operationalise the well-known Janzen-Connell distribution transient pattern (Fig. 2), allowing for quantitative comparisons between different model scenarios. Hence, the presented analyses identify several links between negative plant–soil feedbacks and emergent spatial seedling distribution patterns, and how these links may depend on tree species traits as well as processes occurring in the soil environment.

First, our findings suggest that plant–soil feedbacks acting through seed decay, thus possibly due to soil auto-toxicity, create relatively steep gradients (i.e. high amplitude) of increasing seedling density away from the parent tree (Fig. 4). When this type of plant–soil feedback is dominant, the maximum seedling density would be expected at relatively large distances from the parent tree (Fig. 4). In contrast, when plant–soil feedback acts through increased seedling mortality, which is often due to soil pathogens, patterns in the model are less pronounced in amplitude, and also the maximum amplitude is observed closer to the parent tree (Fig. 4). In this case, the smaller amplitude can be partly explained by a larger centre height, as seedlings do emerge close to the parent tree due to continuous seed input and establishment.

Second, we found that gradients of increasing seedling density away from the parent tree were also steeper for species producing farther-dispersing seeds (Fig. 6). In addition, the maximum seedling density would be expected to occur farther from the parent tree for farther-dispersing species (Fig. 6). Here it should be noted that our results are line with previous studies suggesting that Janzen-Connell distributions are only found when the characteristic scale of seed dispersal exceeds the scale over which negative feedbacks develop^50,51^. Given that we focus on feedbacks that occur through the soil, it seems likely that scales over which negative feedback develop are smaller than those including aboveground herbivores, for example^51^. Limited mobility of the inhibiting factor seems a reasonable assumption for many tropical tree species and our modelling approach, while in cases where soil pathogens would disperse farther than seeds, alternative spatial patterns might emerge^52^.

Third, our simulation results suggest that tree species’ position along the growth-defence trade-off axis may be reflected in the spatial distribution pattern of seedlings around their parent tree (Fig. 7). Specifically, we found that tree species investing in growth rather than defence exhibited stronger Janzen-Connell distributions, in that they were characterized by a higher amplitude and lower centre height. In addition, species investing more in growth reached the maximum seedling at smaller distances to the parent tree (Fig. 7). This latter finding is somewhat counterintuitive, as one might expect that faster growing species would escape more easily from their parent tree and reach higher seedling densities farther away. Here it should be noted that we considered that the processes generating negative plant–soil feedback were similar for species along the growth-defence tradeoff axis. Empirical studies suggest, however, that the processes driving feedbacks may differ between slow-growing shade tolerant and fast-growing shade intolerant species (for example^53^).

For the presented model framework, we could establish that Janzen-Connell distributions can only emerge as a transient phenomenon (Fig. 2; see also^35^). This result can at least in part be attributed to the fact that only a single tree species was considered. On the long term, in the absence of competition with other species, a single species would either cover the entire space at uniform density or exclude itself if plant–soil feedbacks are too strongly negative. In the case of species persistence, a constant uniform adult tree density would also yield a stable uniform soil condition, as well as a constant and stable seed rain. Hence, the plant–soil feedback strength would be in equilibrium, yielding a constant, spatially uniform seedling density as well. Would be of interest to broaden the set of initial conditions studied, considering multiple adults trees, and to consider temporal disturbances that would create gaps in the adult tree canopy cover. For these situations, novel spatial behavior might be observed, for example due to interactions between multiple travelling wave fronts^52,54^.

Several further explorations would be of interest. The model could be expanded to take explicit consideration of multiple timescales, and study the dynamics of cohorts of seeds that are produced annually (e.g.^55^). Given the relatively short lifespan (i.e. less than one year) of the vast majority of tropical tree seedlings (e.g.^56^), cohort-based simulations may exhibit lower centre heights and hence larger amplitudes for the case of increased seed mortality as well. Whether such model adjustment would affect the distance at which the highest seedling density can be found is currently unclear, and would warrant further research. Another interesting analysis would be to explore the differences in the spatial distribution of tropical tree seedlings, whose dispersal characteristics are relatively well known for a considerable number of species (e.g.^57^). Furthermore, as the current model includes both mechanisms (growth inhibition and increased mortality) in a single variable with a uniquely defined diffusion coefficient—which in reality would probably assume very different values for autotoxicity and soil-borne pathogens—considering two separate equations for each individual mechanism may represent a valuable extension of our model. Finally, the model could be used to further explore the spatial distribution patterns of seedlings emerging from different relationships between plant traits related to growth/defence tradeoff and the mechanisms of tree density-dependent feedbacks.

Once the spatial dynamics for a single tree population have been analysed, a further natural expansion of the model framework would be to consider multiple species. This extension seems particularly relevant within the context of hyperdiverse tropical forest communities. A particular strength of theoretical models is that they can explicitly quantify the effect of interspecific interactions by performing simulation experiments that consider a species’ dynamics both in isolation, and in a multispecies context^34^. Such a straightforward comparison would be difficult to find or create in real forest ecosystems. Important outstanding questions that could be addressed with this extended framework related to: (1) the extent to which Janzen-Connell distribution patterns become stronger or weaker in multispecies systems with multiple species; (2) the extent to which Janzen-Connell distribution patterns are transient phenomena or whether temporal stability of these patterns would be observable in multispecies systems.

In addition, the phenomenological modelling approach that was adopted in this study could provide the starting point for the development of a process-based model that aims to describe tropical forest ecosystems at a higher level of representational detail. For example, it has been suggested that plant–soil community interactions could be considered more mechanistically by integrating these interactions within a resource competition framework^58,59^. The spatially explicit modelling approach utilized here could then also be extended to explicitly model belowground biomass distributions and its impact on the availability of resources such as soil nutrients and water^29,52^. Such an extended model analysis may reveal that alternative mechanisms (e.g. involving intraspecific resource competition) could drive similar patterns as the ones driven by negative plant–soil feedback. Moreover, such an extended model may identify specific constraints, in terms of environmental and resource conditions, under which negative plant–soil feedback can operate as a driver of spatial organization^52^. However, to fully resolve the relative importance of different mechanisms for driving the dynamics of a specific tropical forest ecosystem, a closer connection between observational data and model output may be needed. Within this context, moving from the partial differential equation formalism to an individual-based model framework (e.g.^34,60^). Then, the model output would consist of point patterns that could be analyzed using spatial statistics (e.g.^61^) to infer strengths of density-dependent mechanisms (e.g.^62,63^). A potential disadvantage of such extended model frameworks is the reduced analytical tractability, and the reduced ability to infer causal mechanisms and attribution of emerging patterns to underlying processes (^48,49^). Hence, the prior development of stylized models explicitly focusing on the impact of distinct mechanisms in isolation, as performed in this study, may provide a useful stepping stone toward the development and interpretation of more detailed process-based models of forest ecosystems. The large number of tree species occurring within forest communities motivates the search for density-dependent mechanisms that stabilize multispecies dynamics^64^. Negative plant–soil feedback has been identified as a promising explanation for the maintenance of plant biodiversity in general^23,65–67^ and forest biodiversity in particular^7,12,22,24^. There is considerable discussion in the scientific literature, however, regarding the methods used and challenges involved in inferring the strengths of these feedbacks from observational data (e.g.^68–71^). Part of this discussion revolves around the question whether correlations between adult and seedling density at a particular spatial scale (i.e. a (selected sub-region within a) field plot) provide the information needed to calculate the strength of density-dependent feedback. The development of spatially explicit frameworks that simulate seedling distribution patterns, given the distribution of adult trees and hypothesized strengths of plant–soil feedback, would generate more explicit hypotheses that would utilize additional information provided by observational field data. Our model analysis provides a first step towards the development of such a theoretical framework, linking different types of plant–soil feedback to emergent spatial distribution patterns of tree seedlings.

## Figures and Tables

**Figure 8:**
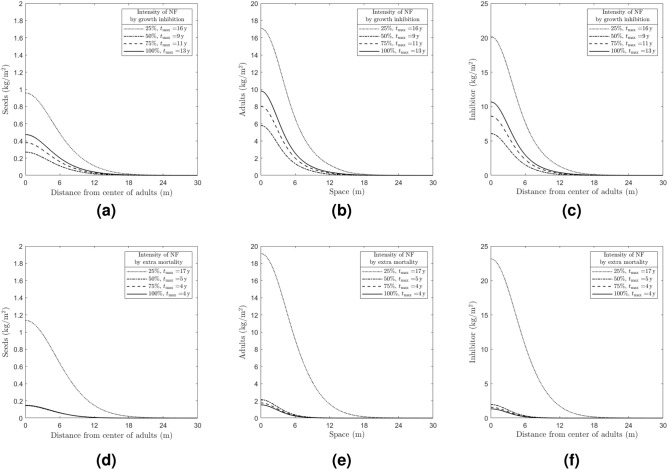
Numerical investigation of the NF influence on the emergence of Janzen-Connell distributions by means of exclusively growth inhibition (top row) and increased mortality (bottom row) for *S* (**a,d**), *A* (**b,e**), and *I* (**c,f**). The plots show the variables’ profiles at time $t=t_{\rm{max}}$ (where the maximum amplitude for *N* is reached, indicated in the legend) obtained by simulating System (1) for different values of the establishment sensitivity to autotoxicity parameter $r_T$, corresponding to different percentages of $r_T^{{\textrm ref}}=68 \, {\textrm m}^2 \, {\textrm kg}^{-1}$ (top row), and different values of $r_P$ representing the increased mortality induced by soil-borne pathogens, corresponding to different percentages of $r_P^{{\textrm ref}}=2 \, {\textrm m}^2 \, {\textrm kg}^{-1} \, {\textrm year}^{-1}$ (bottom row). The other parameter values are fixed as given in “Numerical setup”. The corresponding *N* profiles are shown in Fig. 4.

**Figure 9:**
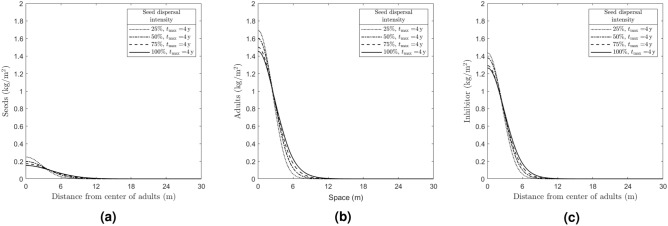
Numerical investigation of the seed dispersal influence on the emergence of Janzen–Connell distributions for *S* (**a**), *A* (**b**), and *I* (**c**). The plots show the variables’ profiles at time $t=t_{\rm{max}}$ (where the maximum amplitude for *N* is reached, indicated in the legend) obtained by simulating System (1) for different values of the seed dispersal coefficient $d_S$, corresponding to different percentages of $d_S^{{\textrm ref}}=3 \, {\textrm m}^2 \, {\textrm year}^{-1}$ (the other parameter values are fixed as in “Numerical setup”). The corresponding *N* profile is shown in Fig. 6.

**Figure 10:**
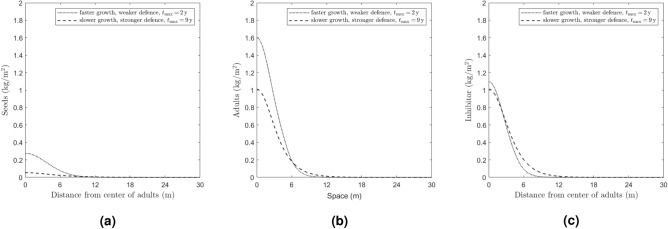
Numerical investigation of the influence of different growth/defence approaches on the emergence of Janzen–Connell distributions for *S* (**a**), *A* (**b**), and *I* (**c**). The plots show the variables’ profiles at time $t=t_{\rm{max}}$ (where the maximum amplitude for *N* is reached, indicated in the legend) obtained by simulating System (1) for a species with faster growth, weaker defence ($g_A=0.9 \, {\textrm year}^{-1}$ and $r_P=2 {\textrm m}^2 \, {\textrm kg}^{-1} \, {\textrm year}^{-1}$, dotted line) and slower growth, stronger defence mechanisms ($g_A=0.02\, {\textrm year}^{-1}$ and $r_P=0.1 \, {\textrm m}^2 \, {\textrm kg}^{-1} \, {\textrm year}^{-1}$, dashed line), respectively (the other parameter values are fixed as in “Numerical setup”). The corresponding *N* profile is shown in Fig. 6.

## Data Availability

The datasets generated and/or analysed during the current study are available in the Github repository, https://github.com/aiuorio28/PSNF-Scientific-Reports.

## Appendix A: Parameter calibration

In the following we describe how we obtained the ecologically feasible values/ranges for the model parameters in (1).

- $A_{\rm{max}}$ (the adult tree carrying capacity) is estimated from Barro Colorado Island (BCI) data reporting the total aboveground biomass densities of $28 -30\, {{\textrm kg}}\, {{\textrm m}}^{-2}$ in^41^. We rounded this up to $30\, {{\textrm kg}}\, {{\textrm m}}^{-2}$.
- $g_S$ (the seed growth rate) is computed as the ratio $g_S=\frac{g_S^{sp} \, g_S^{bc}}{A_{\rm{max}}}$, where $g_S^{sp}$ is the average seed production and $g_S^{bc}$ is the average seed weight. According to the data presented in^72^, $g_S^{sp}$ is around 0.15 seeds ${\textrm year}^{-1} \, {\textrm mm}^{-2}$, which corresponds to $15 \times 10^{4}$ seeds ${{\textrm year}}^{-1} \, {{\textrm m}}^{-2}$. The mass of a single seed varies between $0.002-1\, {{\textrm g}}$^73–75^. Therefore we obtain
  7 $$\begin{aligned} g_S = \frac{15\cdot 10^{4}\cdot (0.002-1)\,\cdot 10^{-3}}{30}\, {\textrm year}^{-1} = (0.01 - 5)\, {{\textrm year}}^{-1}. \end{aligned}$$
- $k_S$ (seeds’ turnover rate) is computed as an average total seed turnover rate of 1-6 ${{\textrm year}}^{-1}$ based on a review of seed longevity as observed in tropical forests globally^76^.
- $g_N$ (the transition rate from seeds to seedlings) is given by the product of two factors $g_N = g_N^{tp} \cdot g_N^{bc}$, where:
  - The seed-to-seedling transition probability $g_N^{tp}$ is calculated using BCI data as follows: $1-7\%$ of seeds in the seed bank germinates after gap formation^77^. Also, there is a difference of approximately 2-3 orders of magnitude between seed production and seed germination, which quickly follows production^78^. Therefore, we can conclude that $1\%$ of seeds typically germinates. Assuming a maximum of $60\%$ reduction of seedlings due to density-dependent effects, and that $1\%$ germination is observed after these effects occurred, we obtain $g_N^{tp} = 0.01/0.4 = 0.025\, {{\textrm year}}^{-1}$. (Note: We expect this value to be smaller than $k_S$.)
  - A biomass conversion factor $g_N^{bc}$ due to the increase in biomass coinciding with the transition from seed to seedling . This conversion factor is given by the ratio between the biomass of a seedling and the biomass of a seed. The former is estimated to be 10 g on average (e.g.^79,80^); i.e. $g_N^{bc}=\frac{10}{(0.002-1)}=10-5000$.
  Consequently, we have that
  8 $$\begin{aligned} g_N = 0.025 \cdot (10-5000) \,{{\textrm year}}^{-1} = 0.25 - 125 \,{\textrm year}^{-1}. \end{aligned}$$
- $\beta$ and $r_T$ (establishment sensitivity to toxicity parameters) correspond to parameters $\beta$ and $\gamma$ in^6^, respectively. Therefore, $\beta =10^{-5}$ and $r_T \in [0,68]$ (mimicking woody plants).
- $k_N$ (death rate of the seedlings) is based on^18^, according to which $2-74\%$ of the seedlings have died after two months. Therefore, $k_N \in [0.02, 0.74]$.
- $r_P$ (the increased mortality of seedlings due to the inhibitor) is obtained by assuming that when *I* reaches its equilibrium values $I^*$ (see “Appendix B”) the mortality increases by a factor $X*k_N$, with $0.3<X<0.9$^81, 82^. Since the absolute maximum increased mortality observed in empirical studies, i.e. the reduction that happens when *I* reaches the theoretical maximum toxicity level *I* ($=A_{\rm{max}}$ with our parameter choice), is 90% (e.g.^83^), we get $0.2< r_P < 0.7$.
- $g_A$ (the transition rate from seedlings to adults is given by the relation $g_A = g_{SS}^{tp} \cdot g_{SA}^{tp} \cdot g_A^{bc}$, where
  - The seedling-to-sapling transition rate $g_{SS}^{tp}$ is $0.0025-2\%$ according to^75^.
  - The sapling-to-adult transition rate is estimated as $0.015-0.1\%$, based on^84–86^.
  - The seedling-to-adult biomass conversion rate $g_{SA}^{tp}$is given by the ratio between the biomass of a sapling and the one of an seedling. In particular, the sapling biomass is assumed to span between 1 and 10 ${{\textrm kg}}$, with a median value of 5 ${{\textrm kg}}$^87–89^.
  Therefore, we have
  9 $$\begin{aligned} g_A = \left[ (0.000025 - 0.02) \cdot (0.015-0.1)\cdot 5 / 0.01 \right] \, {{\textrm year}}^{-1} = 0.00019 - 1\, {{\textrm year}}^{-1}. \end{aligned}$$
- $c_A$ (the growth rate in adults’biomass density) is calculated by imposing that the maximum growth rate (given by $\frac{c_A\,A_{\rm{max}}}{4}$, i.e. the logistic function $c_A \, A\,\left( 1-\frac{A}{A_{\rm{max}}} \right)$ evaluated at the maximum $A=\frac{A_{\rm{max}}}{2}$) is equal to 2 ${{\textrm kg}} \, {{\textrm m}}^{-2} \, {{\textrm year}}^{-1}$^90,91^. This gives $c_A = \frac{4}{15} \, {{\textrm year}}^{-1}$, which can be rounded off to $0.25\, {{\textrm year}}^{-1}$.
- $k_A$ (the mortality rate of adult trees) is assumed to be proportional to the inverse of the average longevity of an adult, which is approximately $100\, {{\textrm year}}$^92,93^. We then consider $k_A=0.01\, {{\textrm year}}^{-1}$.
- $c_T$ (the growth rate of inhibitor density due to adult tree density) is computed based on the ecologically reasonable assumption that the response time in the presence of an adult tree biomass is relatively rapid (order of half a year to year before soil equilibrates, see e.g.^94^). In average, we then get $c_T = 0.7\, {{\textrm year}}^{-1}$. We observe that $c_T>k_A$, since toxicity increases also when adults are living.
- $k_I$ (the toxicity decay rate), analogously to $c_T$, is calculated based on the assumptions that the pathogens effect disappears quite quickly (around half a year to a year) after adult trees are removed. Therefore, we consider $k_I = 0.7\, {{\textrm year}}^{-1}$.
- $d_S$ (the diffusion coefficient for seeds) was derived using empirically derived seed dispersal kernels^95^. Specifically, using we first simulated seed distribution patterns around a single adult tree based on a typical empirical seed dispersal kernel^95^, and then approximating this distribution by a Gaussian. This Gaussian distribution could then be reproduced with a diffusion model, selecting the diffusion coefficient that provided the best fit to this Gaussian distribution. Through this procedure, we obtained best-fitting values within the range $d_S = 3-4 \, {\textrm m}^2 \, {\textrm year}^{-1}$.
- $d_I$ (the diffusion coefficient of toxicity) this spatial parameter is more challenging to parameterize than $d_S$, as direct observations are not available (in contrast to observed seed dispersal patterns). Hence, our general approach is to vary $d_I$, while keeping all other parameters fixed, to identify the range for which JC distributions emerge. To set the upper bound of the range to be considered, we relied on previous studies suggesting that negative density-dependent effects occur within a range of 30 m around adult trees^11,15,22^.

## Appendix B: Steady-states

The steady-states of System (1) are given by the solutions to the following system

10a $$\begin{aligned} 0&= g_S \cdot A - k_S \cdot S, \end{aligned}$$

10b $$\begin{aligned} 0&= \frac{g_N \cdot S}{1+\beta \cdot e^{r_T \cdot I}} - \left( k_N + g_A \left( 1-\frac{A}{A_{\rm{max}}} \right) + r_P \cdot I \right) \cdot N, \end{aligned}$$

10c $$\begin{aligned} 0&= \left( g_A \cdot N + c_A \cdot A \right) \cdot \left( 1-\frac{A}{A_{\rm{max}}} \right) - k_A \cdot A, \end{aligned}$$

10d $$\begin{aligned} 0&= c_T \cdot A - k_I \cdot I. \end{aligned}$$

Because of the complexity deriving by the exponential term in the denominator of Eq. (10b), this system has been carefully studied in its corresponding nondimensional form in^35^, to which we refer for further details. For the purpose of the current paper, however, we merely recall that System (10) admits two solutions given by

11a $$\begin{aligned} E^*_0&= \left( S^*_0,N^*_0,A^*_0,I^*_0 \right) = \left( 0,0,0,0 \right) , \end{aligned}$$

11b $$\begin{aligned} E^*_1&= \left( S^*_1,N^*_1,A^*_1,I^*_1 \right) = \left( \frac{g_S \cdot A^*}{k_S}, \, \frac{g_S \, f \left( \frac{A^*}{k_I} \right) }{k_S \, \left( k_N + \frac{r_P}{k_I} \, A^*+ g_A \, \left( 1-\frac{A^*}{A_{\rm{max}}} \right) \right) } \, A^*, \, A^*, \, \frac{c_T \cdot A^*}{k_I} \right) , \end{aligned}$$

where $A^*$ is the unique solution of

$$\begin{aligned} f \left( \frac{A}{k_I} \right) \, A = g(A) \, A, \end{aligned}$$

with

$$\begin{aligned} f(X)&:=\frac{g_N}{1+\beta \cdot e^{r_T \cdot X}}, \\ g(X)&:= \frac{k_S \left( c_A \left( \frac{X}{A_{\textrm max}}-1\right) +k_A\right) \left( g_A \cdot X-A_{\rm{max}} \, \left( g_A+\frac{r_P \cdot X}{k_I}+k_N\right) \right) }{g_A \cdot g_S \, \left( X-A_{\rm{max}}\right) }. \end{aligned}$$

## Appendix C: Additional plots for *S*, *A*, and *I*

See Figs. 8, 9 and 10.
